# Physiological and comparative proteomic analysis provides new insights into the effects of shade stress in maize (*Zea mays* L.)

**DOI:** 10.1186/s12870-020-2264-2

**Published:** 2020-02-05

**Authors:** Jia Gao, Zheng Liu, Bin Zhao, Peng Liu, Ji-Wang Zhang

**Affiliations:** State Key Laboratory of Crop Biology and College of Agronomy, Shandong Agricultural University, Taian, Shandong 271018 People’s Republic of China

**Keywords:** iTRAQ, Photosynthetic characteristics, Proteomic analysis, Shading, Maize

## Abstract

**Background:**

Shade stress, a universal abiotic stress, suppresses plant growth and production seriously. However, little is known regarding the protein regulatory networks under shade stress. To better characterize the proteomic changes of maize leaves under shade stress, 60% shade (S) and supplementary lighting (L) on cloudy daylight from tasseling stage to physiological maturity stage were designed, the ambient sunlight treatment was used as control (CK). Isobaric tag for relative and absolute quantification (iTRAQ) technology was used to determine the proteome profiles in leaves.

**Results:**

Shading significantly decreased the SPAD value, net photosynthetic rate, and grain yield. During two experimental years, grain yields of S were reduced by 48 and 47%, and L increased by 6 and 11%, compared to CK. In total, 3958 proteins were identified by iTRAQ, and 2745 proteins were quantified including 349 proteins showed at least 1.2-fold changes in expression levels between treatments and CK. The differentially expressed proteins were classified into photosynthesis, stress defense, energy production, signal transduction, and protein and amino acid metabolism using the Web Gene Ontology Annotation Plot online tool. In addition, these proteins showed significant enrichment of the chloroplasts (58%) and cytosol (21%) for subcellular localization.

**Conclusions:**

60% shade induced the expression of proteins involved in photosynthetic electron transport chain (especially light-harvesting complex) and stress/defense/detoxification. However, the proteins related to calvin cycle, starch and sucrose metabolisms, glycolysis, TCA cycle, and ribosome and protein synthesis were dramatically depressed. Together, our results might help to provide a valuable resource for protein function analysis and also clarify the proteomic and physiological mechanism of maize underlying shade stress.

## Background

Maize (*Zea mays* L.) as food, feed, and biofuel is one of the most important crops in the world [1]. The photosynthetic rate of maize is relatively high because of C4 cycle, which enriches CO_2_ then raises the carboxylation activity of RuBP [2]. The photosynthesis is influenced by differentiation and development of thin-walled tissue which is extremely sensitive to shading [3, 4]. In recent years, researches pay more attention to shade stress of C4 plants [4–7].

In these conditions (e.g., wet weather, plant density, and altitude), maize is often subjected to low-light stress or self-shading, especially in the later growth stages in the North China Plain, and the decrease of sunshine hours and solar radiation severely restricts maize production [8–10]. The chloroplast, the site of photosynthesis, is very sensitive to external environments, and its structure directly affects photosynthesis [11–13]. Shade stress destroys chloroplast ultrastructure, and reduced chlorophyll synthesis, carbon dioxide fixation and photosynthetic capacity [14–18]. In addition, shade stress induces more superoxide, H_2_O_2_ and hydroxyl radicals in plants [19]. Plants alter morphological and nutrients distribution to accommodate lack light, such as adapting to low-light environments by synthesizing more chlorophyll as part of the light-harvesting complex [20–22]. Increased light results in a denser thylakoid layer structure, and significantly enhanced photosystem I and II (PSI and PSII) activity [23, 24]. Plants have complex physiological and biochemical responses to environmental stress [25, 26]. Therefore, it is important to understand proteomics changes caused by shade stress.

Proteins as direct participants are involved in growth, development, reproduction, metabolism, among other processes, which reflects the response of various physiological functions stimulated by the environment [27, 28]. Differential proteomics focuses on screening and identifying differences and changes in the proteome between different species or states, revealing and validating proteomics changes [29]. Isobaric tags for relative and absolute quantification (iTRAQ) combined with tandem mass spectrometry and multidimensional liquid chromatography provides good accuracy and repeatability, particularly for low levels of protein [1, 30].

Previous studies on maize photosynthesis under shading were limited to the level of apparent physiology, and few studies on comparative proteomics. To gain a systematic and in-depth understanding of the response of maize leaves to different light intensity, and to understand changes in their proteomics, we determined differences in protein abundant in maize leaves under varying light conditions. Together, our study may help clarify the mechanisms of shade stress and its effect on photosynthesis in maize leaves.

## Results

### Grain yield and yield components

Grain yields significantly decreased with respect to the control (CK) after shading (Table 1). During 2015 and 2016, grain yield in shading (S) treatment decreased by 48, and 47%, and supplementary lighting (L) increased by 6 and 11%, respectively. Different light conditions after tasseling affected the yield components. Grains per ear, ear number, and 1000-grain weight decreased in S treatment compared to the CK, resulting in the loss of yield.

**Table 1:** Grain yield and yield components under different light intensities after tasseling from 2015 to 2016

Year	Treatment	Yield (kg ha^− 1^)	1000-grain weight (g)	Grains per ear	Harvest ear number (ears ha^− 1^)
2015	S	6087b	313b	327b	59506b
	CK	11642a	316b	558a	65965a
	L	12314a	333a	560a	66167a
2016	S	6798c	342b	322c	61728b
	CK	12852b	364a	550b	64198a
	L	14278a	367a	590a	66049a

S: 60% shade; L: supplementary lighting on cloudy daylight; CK: ambient sunlight. Values fallowed by a different small letter within a column are significantly different at 5% probability level

### Leaf photosynthetic performance

#### Gas exchange parameters of functional leaves

The photosynthetic rate (*P*_*n*_), transpiration rate (*T*_*r*_), and stomatal conductance (*g*_*s*_) under shade stress were lower than those in controls at the corresponding growth stages (Table 2). The *P*_*n*_, *T*_*r*_, and *g*_*s*_ values decreased by 53, 67, and 68% at VT20 (20 d after tasseling), and those of VT40 (40 d after tasseling) decreased by 67, 59, and 67%, respectively. The *P*_*n*_, *T*_*r*_, and *g*_*s*_ values in L treatment increased by 3, 5, and 15%, and by 8, 15, and 19% at VT20 and VT40 compared to CK, respectively. The intercellular CO_2_ concentration (*C*_*i*_) in S treatment increased by 11 and 10% at VT20 and VT40, respectively; that in the L treatment decreased by 13 and 4% at VT20 and VT40, respectively.

**Table 2:** Photosynthetic gas exchange parameters in functional leaf under different light intensities after tasseling in 2015 and 2016

Year	Stage	Treatment	*P*_n_	*T*_r_	*g*_s_	*C*_i_
2015	VT	CK	45.2	6.8	523.2	135.2
	VT20	S	10.7b	1.0b	74.1c	123.5a
		CK	34.2a	4.4a	245.7b	113.3b
		L	34.6a	4.3a	286.7a	98.0c
	VT40	S	6.3c	0.8b	55.7c	170.3a
		CK	18.7b	2.7a	156.3b	158.0b
		L	20.2a	3.1a	201.3a	146.3c
2016	VT	CK	42. 8	7.4	542.1	149.2
	VT20	S	20.1b	2.3b	93.8b	121.5b
		CK	32.0a	5.4a	280.1ab	108.3b
		L	33.7a	6.1a	319.2a	94.2a
	VT40	S	7.2c	1.1b	51.5b	165.8a
		CK	22.0b	2.1a	171.3a	148.5b
		L	23.7a	2.4a	187.0a	149.2b

S: 60% shade; L: supplementary lighting on cloudy daylight; CK: ambient sunlight; VT: Tasseling stage; VT20: 20 d after tasseling; VT40: 40 d after tasseling; *P*_*n*_: Photosynthetic rate; *T*_*r*_: Transpiration rate; *g*_*s*_: Stomatal conductance; *C*_*i*_: Intercellular CO_2_ concentration. Values fallowed by a different small letter within a column are significantly different at 5% probability level

#### Chlorophyll soil–plant analyses development (SPAD) values

**Fig. 1:**
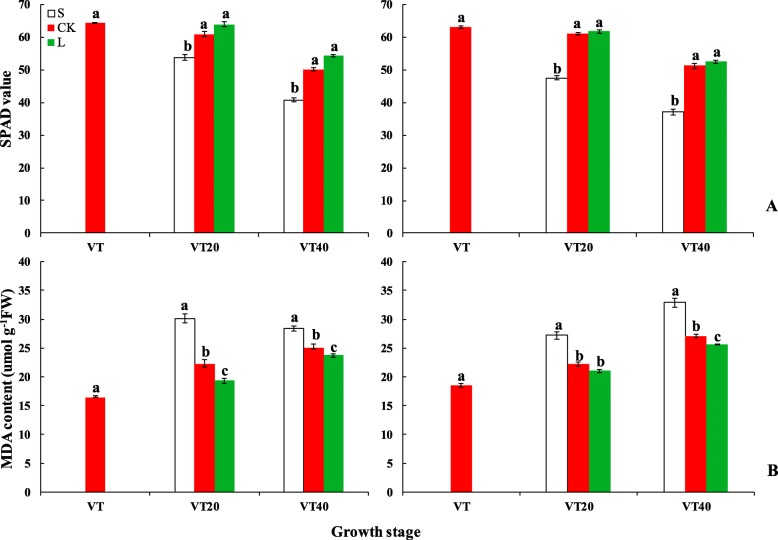
Histograms of SPAD value (**a**) and MDA content (μmol g^− 1^FW, **b**) in functional leaves under different light intensities after tasseling in 2015 and 2016

The effects of different light intensities after tasseling on the chlorophyll SPAD value was differed (Fig. 1a). The SPAD in S treatment decreased by 17 and 23% compared to CK at VT20 and VT40, respectively, whereas that in L treatment increased by 3 and 6% during the same periods.

#### Malondialdehyde (MDA) content of functional leaves

The MDA content of S treatment increased by 29 and 17% at VT20 and VT40, respectively, compared to that of CK; L treatment decreased by 9 and 6%. The application of different light intensities after tasseling had different effects on the senescence characteristics of maize (Fig. 1b).

#### iTRAQ analysis of differential abundance protein species (DAPS) in maize leaves

**Fig. 2:**
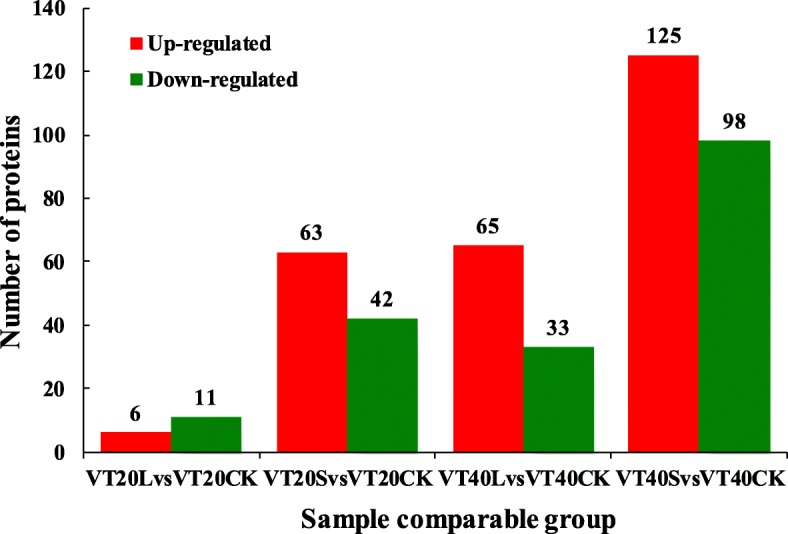
Number of differentially expressed protein identified in S and L treatment

3958 proteins were identified from the maize leaves by MS/MS, of which 2745 proteins have relative quantitative information (Additional file 1). According to the recognition criteria for DAPs (fold change ratio > 1.2 and *p* < 0.05), 105 DAPs were identified from the S treatment 20 days after tasseling; of these, 63 proteins were increased and 42 proteins were decreased. In the L treatment, 17 DAPs were identified 20 days after tasseling, of which 6 proteins were increased and 11 proteins were decreased. The number of DAPs differed at different time points. At later processing time, 223 DAPs were identified in the S treatment, including 125 increased and 98 decreased proteins; 98 DAPs were obtained from the L treatment, including 65 increased and 33 decreased proteins (Fig. 2).

**Fig. 3:**
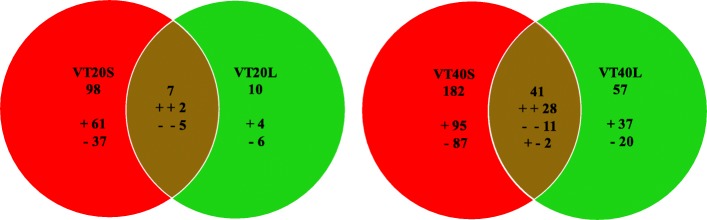
Venn diagrams of differentially expressed proteins that were increased or decreased by different light stress. The “+” and “-"indicate increased and decreased proteins, respectively. The differential expressed proteins in the maize leaves compared with the CK.

Twenty days after tasseling, 98 proteins (61 increased and 37 decreased) and 10 proteins (4 increased and 6 decreased) responded to only the S or L treatment, respectively, whereas 7 proteins were differentially expressed in both S and L treatments. Among these 7 proteins, two were increased under S and L treatments, whereas 5 proteins were decreased under both conditions. Forty days after tasseling, 182 (95 increased and 87 decreased) and 57 (37 increased and 20 decreased) proteins responded to only the S or L treatment, respectively, whereas 41 proteins were differentially expressed in both S and L treatments. Among these 41 proteins, 28 were increased under both S and L treatments, whereas 11 were decreased under both conditions. Two proteins were increased under S treatment and decreased in L treatment (Fig. 3).

#### Functional classification and predicted localization of DAPs

**Fig. 4:**
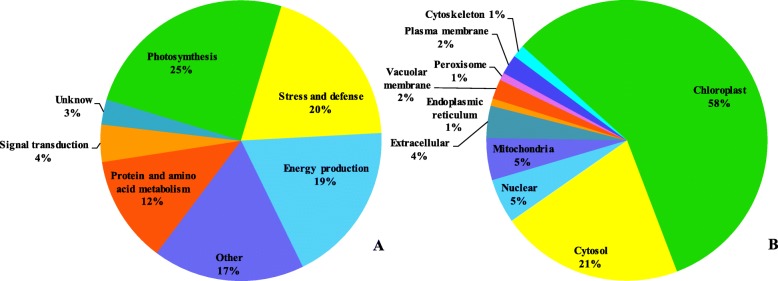
Functional classifications (**a**) and localizations (**b**) of identified proteins

According to the molecular functions listed on the UniProt (http://www.uniprot.org/) and GO websites (http://www.geneontology.org/), the 349 proteins were classified into seven functional categories (Fig. 4a and Additional file 2). These categories were involved in photosynthesis (25%); stress, defense, or detoxification (20%); energy production (19%); protein and amino acid metabolism (12%); signal transduction (4%); other protein functions (17%); and unknown protein functions (3%).

Next, the WoLF PSORT software (http://www.genscript.com/wolf-psort.html) was used to predict subcellular localization. The subcellular localization of the 349 characterized proteins showed that 202 proteins (58%) were located in chloroplasts, 74 proteins (21%) in the cytosol, 18 (5%) in the nucleus, 17 (5%) in the mitochondria, 13 (4%) in extracellular spaces, 8 (2%) in the vacuolar membrane, 8 (2%) in the plasma membrane, 5 (1%) in the cytoskeleton, 3 (1%) in the endoplasmic reticulum, and 3 (1%) in the peroxisome (Fig. 4b). These results indicate that many chloroplast proteins are related to light reactions in maize.

### GO enrichment and KEGG annotation analysis of DAPs

#### GO enrichment analysis of DAPs

**Fig. 5:**
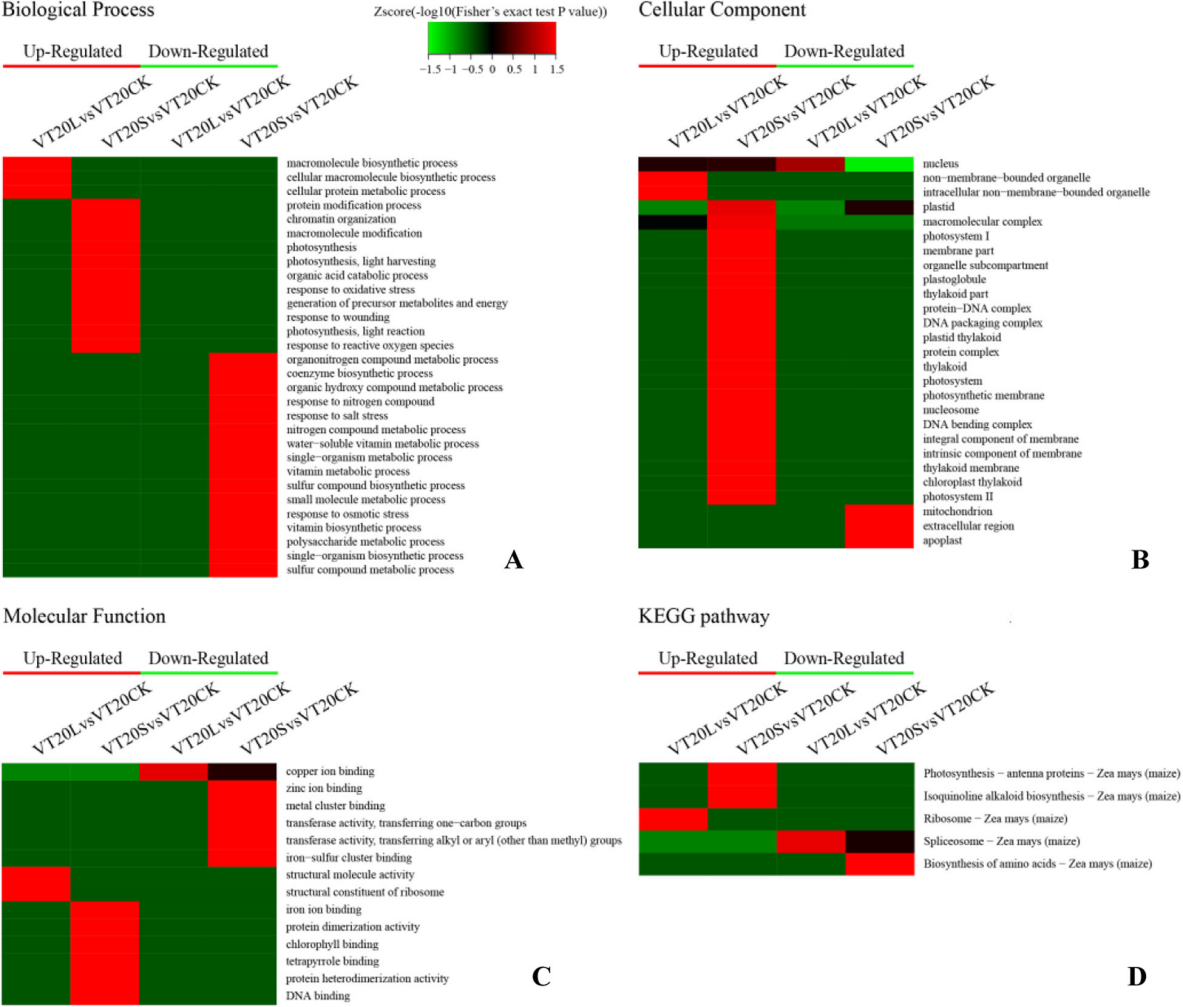
Heat maps obtained from GO and KEGG pathway comparing the protein expression patterns of VT20S, VT20L, and VT20CK. **a** Biological process analysis; **b** Cellular component analysis; **c** Molecular function analysis; **d** KEGG pathway analysis. Red indicates higher expression, green indicates lower expression, and black indicates the same expression levels

**Fig. 6:**
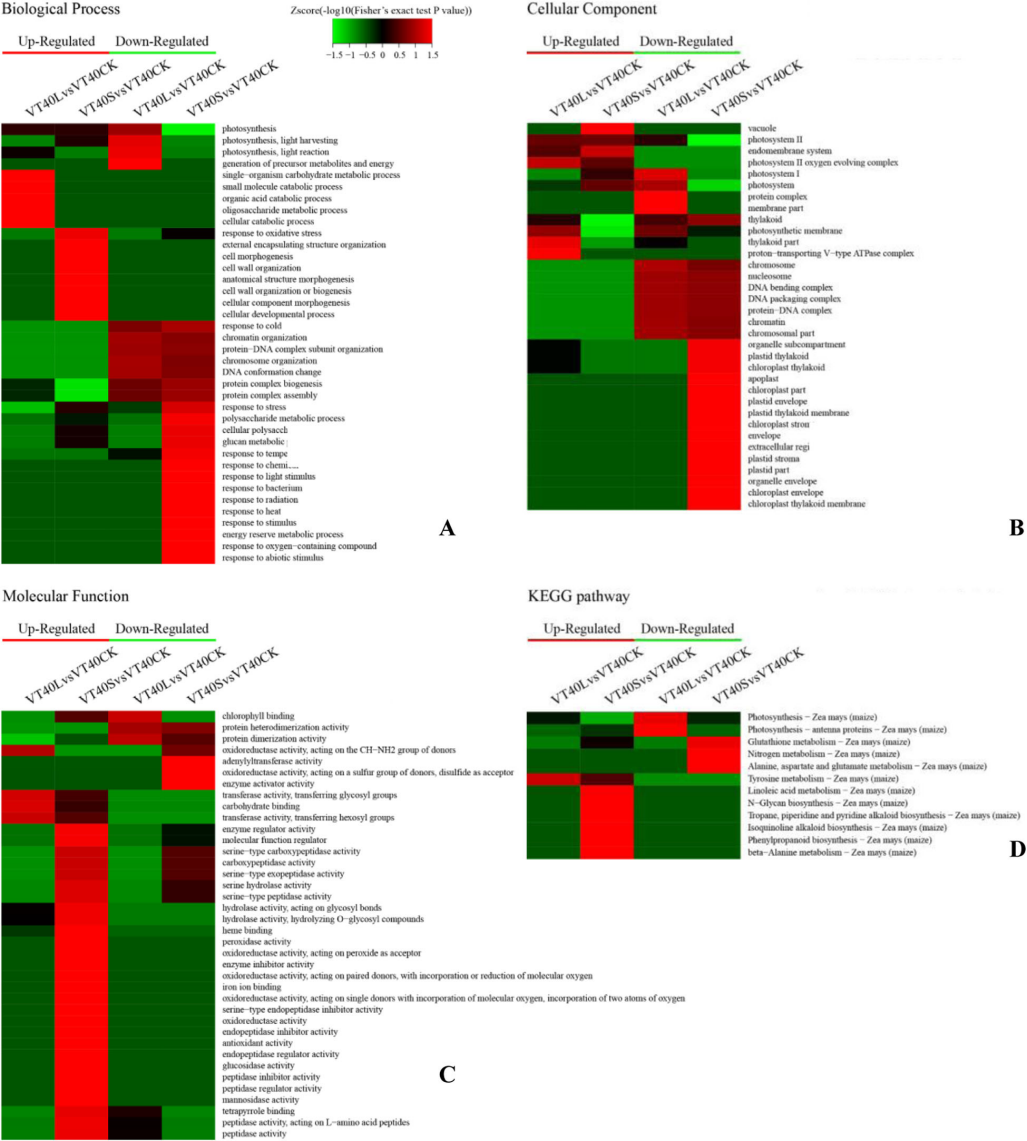
Heat maps obtained from GO and KEGG pathway comparing the protein expression patterns of VT40S, VT40L, and VT40CK. **a** Biological process analysis; **b** Cellular component analysis; **c** Molecular function analysis; **d** KEGG pathway analysis. Red indicates higher expression, green indicates lower expression, and black indicates the same expression levels

To further understand the nature of the identified and quantified proteins (fold change ratio > 1.2 and *p* < 0.05), we annotated their functions and features using GO. The DAPs were grouped into three hierarchically structured GO terms: biological process (Figs. 5a and 6a), cellular component (Figs. 5b and 6b), and molecular function (Figs. 5c and 6c). Go cluster analysis showed that the increased DAPs (VT20L vs. VT20CK) were highly enriched in macromolecule biosynthetic and protein metabolic process, and mainly located in the cell membrane, which play a role in structural constituent of ribosome and structural molecule activity. The increased DAPs (VT20S vs. VT20CK) were mainly involved in photosynthesis, response to oxidative stress and wounding, and protein modification process, and mainly located in the photosystem, which molecular function is the chlorophyll binding. The decreased DAPs (VT20S vs. VT20CK) were mainly involved in redox reactions, carbohydrate metabolism, and other metabolic processes (Fig. 5). This result suggests that the increased DAPs (VT20S) were mainly located in the photosynthetic system and thylakoid structure, and were involved in biological processes such as photosynthesis and protein modification, mainly in “binding” and “signal transduction”. And the DAPs (VT20L vs. VT20CK) were mainly involved in protein metabolic process (Fig. 5).

Compared to the control (VT40Ck), the increased DAPs (VT40S) were highly enriched in cell morphogenesis, oxidative stress reactions and stress defense, and mainly located in the photosystem and vacuole, and the DAPs in the S treatment showed strong enrichment in enzyme regulator activity and oxidoreductase activity. The decreased DAPs (VT20S vs. VT20CK) were mainly located in the plastid and chloroplast and involved in protein complex assembly, stress defense and polysaccharide metabolic process. When the light treatment was applied for 40 days, the increased DAPs were mainly involved in the processes of cellular catabolic, oligosaccharide metabolic and organism carbohydrate metabolic. The decreased DAPs were mainly involved in photosynthesis, energy metabolism, and protein synthesis processes (Fig. 6).

#### KEGG enrichment analysis of DAPs

The KEGG database was used to identify enriched pathways using a two-tailed Fisher’s exact test to test the enrichment of DAPs against all identified proteins. A pathway with a corrected *p*-value < 0.05 was considered significant. These pathways were classified into hierarchical categories according to the KEGG pathway website (http://www.genome.jp/kegg/pathway.html). Twenty days after tasseling, KEGG cluster analysis showed that the DAPs in the L treatment were highly enriched in protein synthesis and shear functions, and the DAPs in the S treatment showed strong enrichment in photosynthesis and amino acid synthesis (Fig. 5d). Forty days after tasseling, KEGG cluster analysis showed that the DAPs in the L treatment were enriched in photosynthesis and tyrosine metabolism, and that DAPs in the S treatment were expressed in nitrogen metabolism, glutathione, alanine, aspartic acid, and glutamic acid metabolism, linoleic acid metabolism, N-sugar chain synthesis (Fig. 6d).

#### Transcriptional expression analysis by quantitative real time polymerase chain reaction (qRT-PCR)

**Fig. 7:**
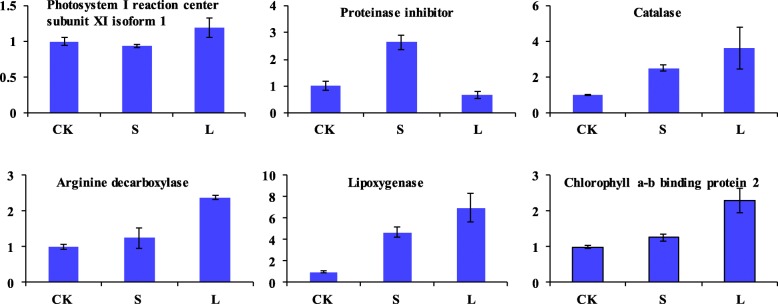
Analysis of transcript levels of the differential abundance protein species between S and L at 20 days after tasseling by qRT-PCR

qRT-PCR results showed that photosystem I reaction center subunit XI isoform 1, proteinase inhibitor, Catalase, arginine decarboxylase and lipoxygenase these five gene transcription level displayed the same trend with the abundance of the corresponding protein species (Fig. 7). In contrast, the chlorophyll a-b binding protein 2 showed the opposite trend at VT20L.

## Discussion

In recent years, rainy weather occurred frequently during the later growth periods of maize, which inhibited maize pollination and decreased yield [31]. Transcriptomics studies showed that many pathways were greatly induced by shade, e.g. DNA synthesis/chromatin structure, light signaling, light reaction and RNA regulation of transcription [32, 33]. However, mRNA expression levels do not fully predict the corresponding protein abundance resulting from post-transcriptional modification and translation regulation [34]. Research on proteomics is helpful to reveal complex changes in maize leaves under shade stress and provide new information concerning the maize response to shade stress in field. We successfully identified 3958 proteins (Additional file 1), which are more than that found by two-dimensional electrophoresis-based proteomics.

### Effects of light intensity on grain yield and photosynthetic properties

No significant difference was observed on microclimatic indexes between treatments, except light intensity, which was monitored periodically (Table 3), and the same results were reported by Du [35]. MDA content often reflects the degree of lipid peroxidation in the body, indirectly reflects the extent of cell damage and leaf senescence, and affects the photosynthesis [36, 37]. Our results indicated that shade after tasseling increased MDA concentration and accelerated leaf senescence, while supplementary lighting delayed the leaves senescence by improving the protective enzymes activity and increasing the ability to scavenge reactive oxygen, as reported by previous researches [24]. Shade after tasseling decreased chlorophyll content, photosynthetic rate, and grain yield resulting from free radical damage in the membrane system [37], whereas supplementary lighting improved photosynthetic capacity and production.

**Table 3:** Microclimate in experimental field under different light treatments

Treatment	Air speed (m s^− 1^)	Air temperature (°C)	Soil temperature (°C)	Relative humidity (%)	Light intensity (μmol m^− 2^ s^− 1^)	CO_2_ concentration (μmol mol^− 1^)
S	0.88a	25.83a	23.28a	47.3a	712.0b	326.48a
CK	0.94a	25.42a	24.55a	51.7a	1675.3a	318.28a

S: 60% shade; CK: ambient sunlight. Values fallowed by a different small letter within a column are significantly different at 5% probability level

### Effects of light intensity on photosynthesis-related proteins

In this study, we identified 87 DAPs involved in photosynthesis after shade and supplementary lighting (Fig. 4a). Photosynthesis including light reaction and carbon-fixation reaction determined plant productivity and energy efficiency, and was very sensitive to abiotic stress [38, 39]. The VT20S treatment enhanced the expression of Chlorophyll a/b-binding protein (A0A096RF43) to improve photosynthetic electron transport chain operation rate, which provide sufficient restoring force and adenosine triphosphate (ATP) for the dark reaction [34]. L induced the expression of photosystem II reaction center Psb28 protein (K7V1V6, B6TYC5) and promoted water photolysis. Ferredoxin (K7U9U9, B6SP61, A0A096QD90) is the terminal oxidase of the photosynthetic electron transport chain, while cytochrome P450 (A0A096QBU9, A0A096PQR7, A0A096SY33, C0P4G2) acts as a terminal oxidase to accept NADPH electrons and jointly participate in the electron transport [1, 40, 41]. Our results showed that S increased the abundance of ferredoxin, cytochrome P450 and PSI reaction center subunits (B4FUT9, P62596, B6TR16, B6U534, B4G1K9) and VT40L decreased ferredoxin, which may mean that plants improve the photosynthetic electron transport to adapt shade stress, resulting in a low-light stress emergency response, and some basic metabolites such as carbon metabolites need to be adjusted to create a new balance under abiotic stress.

**Fig. 8:**
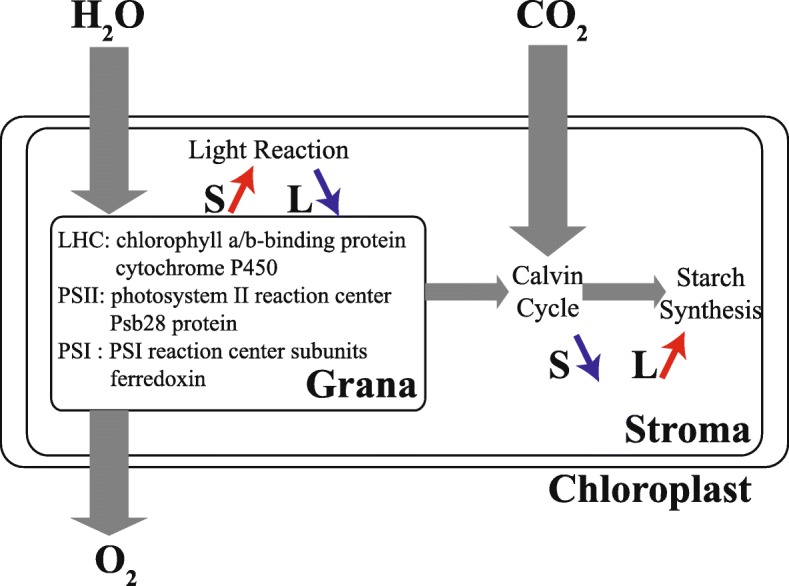
An overview of the photosynthesis processes under different light intensities in maize leaves. Increased is marked by; decreased is marked by

Fructose-bisphosphate aldolase (FBPase), starch synthase and isoflavone reductase are closely related to the sucrose synthesis and photosynthetic products formation [42–44]. In our study, S decreased the abundance of FBPase (B4FR47, A0A096RD67), starch synthase (B8A2L4) and isoflavone reductase (B4FD74) (Fig. 8), which may affect leaf photosynthetic efficiency and decreased ATP and NADPH content. Similarly, Study in leaf ultrastructure suggested that shade destroyed the chloroplast ultrastructure, while supplementary lighting increased the number of grana and lamellae [24]. We speculated that the shading in early grain-filling stage mainly inhibits the PSI and dark reaction, and PSII improves the utilization efficiency of light energy by increasing the abundance of electron transfer-related proteins and reducing the adverse effects of shade on growth and development. PSII-related DAP increased and the CO_2_ fixation and starch synthesis were inhibited in the later growth stage. The photosynthetic mechanism was damaged reflected in gas exchange parameters and relative protein abundance, which decreased biomass and grain yield. Fewer photosynthesis-related proteins were found in VT20L, and more in VT40L. This may be the result of less rainy weather at the beginning of grain-filling, and more rainy weather during the late grain-filling stages. Supplementary lighting promoted the photosynthetic electron transport, enhanced photosynthetic efficiency, and promoted the starch synthesis process. This may explain why the increase in photosynthetic rate and dry matter accumulation in maize (Table 2).

### Effects of different light conditions on energy production-related proteins

Plants decompose complex organic matter into simple compounds via respiratory metabolism, and release energy to maintain plant intermediate metabolites and energy needs [42, 45]. Proteomics analysis showed that polysaccharide degradation-related proteins (1,4-beta-D-glucanase (B4FTK9, B4FPA0), water dikinase (A0A096TN87), 1,3-alpha-glucosidase (K7TGE1, K7VP34)) decreased in S, indicating that the leaves were weakened by degrading various polysaccharides. Similar to these results, shade stress also inhibited the expression of glycolysis-related enzymes, while supplementary lighting has the opposite trend, indicating that shade stress inhibited glycolysis and accelerated plant senescence. The vacuolar ATPase subunit (B4FPE4) continuously increased in senescent leaves, indicating that it is necessary to transport the saccharide output of leaves by the sucrose synthesis or temporarily store it in the cytosol during the aging process. The NADP-dependent quinone oxidoreductase-like proteins (A0A096S7Z2, A0A096U487) in the first complex of mitochondrial respiratory chains were also greatly induced by shade. Their increases may mean that plants can make up for the lack of energy supply by enhancing electron transport and ATP synthesis in the respiratory chain. Beta-galactosidase (A0A096R2T2) is important to produce energy and carbon sources [46]. Alcohol dehydrogenase superfamily protein (K7UAQ8, A0A096SZS3, A0A096TTS5), beta-galactosidase (A0A096R2T2), aconitate hydratase (C0HER4), beta-amylase (A0A0B4J3H2), and other proteins involved in glycolysis and tricarboxylic acid cycle were significantly depressed by shade. Thus, shade destroyed glycolysis, the tricarboxylic acid cycle, and other energy metabolic pathways, which may reduce the energy supply and grain yield. The flexibility of energy metabolism may help to improve the resistance of maize to shade stress.

### Effects of light intensity on stress/defense/detoxification-related proteins

Plants often accumulates a large amount of reactive oxygen species (ROS) and free radicals under stress, which seriously affects plant homeostasis and accelerates plant senescence [47]. Plants also formed defense systems to remove many free radicals and ROS over evolutionary time [48]. Peroxidase (Prx, A0A0B4J3G7, A0A0B4J3A8, B4FVT1, A0A096T686, A0A096SIT0, A0A096RME9), catalase (K7UGM3, A0A0B4J352) and cysteine protease (Cpr, B4FS65, B4FZ79, A0A096S518) were induced by shade and involved in the process of defense and detoxification [49–51]. Prx has stronger reactive oxygen scavenging capacity than other peroxidases (e.g., SOD, POD, CAT, and APX) [49, 50], which enhanced the antioxidant capacity of maize leaves under shade stress. Cpr as a proteolytic enzyme, is involved in the degradation of damaged proteins, which decreased the soluble protein content and enhanced osmotic regulation [51]. Shade stress enhance shade adaptation or resistance in maize leaves by improving the scavenging capacity of ROS, similar results have been found in grapevine leaves after heat stress [42]. Heat shock protein 70 (Hsp70, A0A096R6Z8, C0P732) is an important stress-induced protein and molecular chaperone protein, and also protects and repairs PSII under light suppression [52, 53]. Glutathione S-transferase (GSTs) is involved in metabolism, scavenging free radicals and alleviating oxidative damage [40, 54], which also can detoxify membrane lipid peroxides and oxidized DNA degradation products by binding to reduced glutathione [55]. Thus, increased GST activity may contribute to maize resistance under shade stress.

### Effects of light intensity on protein and amino acid metabolism-related proteins

Protein metabolism generally include protein biosynthesis, protein folding, modification, and degradation [56]. iTRAQ analysis identified 18 proteins related to transcriptional translations, including three nucleic acid binding proteins, 60S, 40S (nuclear protein synthesis), 30S, and 50S (plastid and mitochondrial protein synthesis) ribonucleoprotein (A0A0B4J3C6, C0P9S2) and elongation factors (A0A096PU69, A0A096QDW6, B6TWN7). The elongation factor was depressed by shade, which indicated that shade stress inhibited the transcriptional translation-related proteins and affected the protein synthesis [57, 58]. We also identified 20 proteins associated with protein folding, modification, and degradation. Shade suppressed post-transcriptional protein modification, disulfide exchange regulation and important enzyme activity regulator by inhibiting the expression of Thioredoxin [59]. Shade induced the expression of protein-binding protein (C4J5Y0), which is a ubiquitous coenzyme and enzyme modulator [60]. In this context, different light intensities after tasseling can affect protein folding, modification and degradation, and thus regulate various physiological and biochemical metabolic processes of plants.

Based on our results and previous studies, we could take some strategies to minimize the harm of shading for maize. Firstly, it is possible to enhance the expression of photosynthesis-related proteins through weak light exercise, and improve the photosynthetic electron transport chain operation rate and stress tolerance. Secondly, exogenous hormone regulation may be a convenient way to reduce the negative impact of low light on maize. Lastly, choose the high resistance varieties and adjust the sowing date to avoid rainy weather in the late growing period, to ensure appropriate light conditions which make maize high efficiency photosynthesis. In a word, our results may improve the understanding of the protein expression mechanism in maize leaves under shade stress, and help people to improve the photosynthesis and yields of maize by genetic improvement and agronomic management practices.

## Conclusions

Based on iTRAQ proteomics analyses, 3958 proteins were identified in maize leaves, among them, 349 significant DAPs were related to photosynthesis, defense, energy metabolism, protein synthesis, signal transduction, and other biological processes. Compared with the control, shade induced the expression of photosynthetic electron transport chain related proteins (especially light-harvesting complex) and stress/defense/detoxification, however, the proteins related to calvin cycle, starch and sucrose metabolisms, glycolysis, TCA cycle, and ribosome and protein synthesis were dramatically depressed. The results offer novel insights into the proteomic level and physiological response mechanism of maize under shade stress.

## Methods

### Plant materials and stress treatments

Experiment were conducted at the Shandong Agricultural University Experimental Farm (36°09′N, 117°09′E, 158 m *a.s.l.*) and the State Key Laboratory of Crop Biology, China in 2015 and 2016. This research was approved by Shandong Agricultural University complying with the Convention on the Trade in Endangered Species of Wild Fauna and Flora. The region was characterized by a temperate continental monsoon climate, and the accumulated temperature, rainfall, and artificial lighting time during the study period are listed in Table 4. Additional information about soil at the region is available in Gao et al. (2017a). Maize hybrids, Zhengdan 958 (ZD958, Zheng58/Chang7–2) planted (67,500 plants per hectare) in this study, which approved by the National Crop Variety Examination and Approval Committee of China in 2001. The materials were bought from Tai’an Denghaiwuyue Taishan Seed Industry Co., LTD. Three treatments (S: 60% shade; L: supplementary lighting on cloudy daylight; and CK: ambient sunlight) were arranged in the field. Each experimental plots are 5 60-cm rows of maize wide by 9 m long. Shade and supplementary lighting were applied at the tasseling stage and remained until harvest. Shade tents were constructed with commercially available shade cloth (Hongda Shade Cloth Company, Shouguang, China) and scaffolding that kept the cloth about 2 m above the crop, and the maximum lighting intensity of L on cloudy daylight was 1600–1800 μmol m^− 2^ s^− 1^. Disease, weeds, and pests were well controlled in each treatment. Atrazine and acetochlor were surface-applied before maize germination to control weeds, and phoxim was applied to control corn borers.

**Table 4:** Accumulated temperature and rainfall of summer maize growth periods (June–September) in 2015–2016

Year	Accumulated temperature (°C d)	Rainfall (mm)	Artificial lighting time (h)
2015	3080.2	282.6	213.1
2016	3073.1	439.8	214.3

### Field microclimate

The measure methods for irradiance, canopy CO_2_ concentration, relative humidity, canopy air temperature, wind speed, soil temperatures have been reported in our previous studies [6] (Table 3).

### Gas exchange parameters of functional leaves

The photosynthetic rate (*P*_*n*_), transpiration rate (*T*_*r*_), stomatal conductance (*g*_*s*_), and intercellular CO_2_ concentration (*C*_*i*_) were measured according to the method described by Gao et al. (2017) [7].

### Chlorophyll SPAD values

The SPAD value was measured at VT, VT20, and VT40 using a portable chlorophyll meter (SPAD-502, Minolta Camera Co., Osaka, Japan). Ten plants per treatment were randomly selected for measurements.

### MDA content of functional leaves

Plants were sampled at VT, VT20, and VT40, with three replicates per treatment. For each plant sample, ear leaves were collected and stored at − 40 °C prior to analysis. Functional leaf MDA content was measured according to the thiobarbituric acid method [36], in units of μmol g^− 1^FW.

### Grain yield and yield components

Thirty ears from the middle three rows of each plot were harvested at R6 using a continuous sampling method and then used to determine yield and yield components. Standard moisture content is 14%.

$$ \mathrm{Yield}\ \left(\mathrm{kg}\ {\mathrm{ha}}^{-1}\right)=\mathrm{ears}\ \left(\mathrm{ears}\ {\mathrm{ha}}^{-1}\right)\times \mathrm{kernels}\ \mathrm{per}\ \mathrm{ear}\times \mathrm{thousand}\ \mathrm{grain}\ \mathrm{weight}\ \left(\mathrm{g}/1000\ \mathrm{grains}\right)/1{0}^6\times \left(1-\mathrm{moisture}\ \mathrm{content}\%\right)/\left(1-14\%\right) $$

### iTRAQ proteomics analysis

According to previous studies, we chose the VT20 and VT40 stages as two distinct phases during which to determine the proteins expression changes during the early and late stages of grain filling. We sampled five ear leaves from five plants at the center of each plot at VT20 and VT40. The middle portions of the leaves were collected and frozen in liquid nitrogen and stored at − 80 °C prior to analysis. Each treatment had three biological replicates (Additional file 3). Samples were ground into a fine powder in liquid nitrogen using a mortar and pestle, and then to extract proteins. The extracted protein solutions were digestion by trypsin. Following trypsin digestion, the peptide was desalted, vacuum-dried and iTRAQ labeling. Then the sample were fractionated by high-pH reverse-phase high-performance liquid chromatography fractionation which were performed liquid chromatography–tandem mass spectrometry analysis. The specific analysis method of proteomics was provided by Jingjie PTM BioLabs, Inc. The more detailed method was described in Additional file 4.

The resulting MS/MS data were processed using the Mascot search engine (v.2.3.0, Matrix Science, London, UK). Tandem mass spectra were searched against *Zea mays* database. The search was performed specifying Trypsin/P as a cleavage enzyme, allowing up to two missing cleavages. Mass error was set at 10 ppm for precursor ions and 0.02 Da for fragment ions. Carbamidomethyl on Cys was specified as a fixed modification and oxidation on Met was specified as a variable modification. iTRAQ-8-plex was selected in Mascot for protein quantification. The false discovery rate was adjusted to < 1% and the peptide ion score was set at ≥20. We used CK samples from the same period as a reference, all other samples were compared to CK. To ensure the accuracy of quantitative results, we obtained quantitative protein information from at least two biological replicates before further analysis. The average of three biologic replicates was taken as the final protein abundance, and proteins with average protein abundance that changed by more than 1.2-fold in different stages (*p* ≤ 0.05) were defined as DAPs.

Functional annotations of DAPs species were performed using GO. Based on these annotations, proteins were classified into three categories: biological process, cellular component, and molecular function. Then we used the WoLF PSORT software to predict subcellular localization. We used the KEGG database to predict the main metabolic pathways and the DAPS biochemical signal transduction pathways. Statistical analyses were conducted using analysis of variance (ANOVA) in SPSS 20.0. We assessed differences among treatments using a least significant difference (LSD) test at a probability level of 0.05.

### RNA extraction and quantitative real-time PCR

We selected six identified proteins and analyzed the expression levels of the corresponding genes by qRT-PCR. Total RNA was extracted from leaves using the TRIzol reagent (Aidlab, Beijing, China). Total RNA was treated with DNaseI to remove genomic DNA contamination. The specific primers for the target genes (Additional file 5) were designed by Primer 3.0 software. qRT-PCR was performed using the Bestar SYBR Premix Ex Tag KIT (Germany). qRT-PCR was performed in 20 μl volumes containing 10 μl 2X SYBR Green qPCR master mix, 2 μl cDNA, 0.5 μl of each gene-specific primer and 7 μl ddH_2_O. PCR conditions were: 95 °C for 2 min, 40 cycles of 10 s at 95 °C, 60 °C for 30 s and 72 °C for 30 s, a melt curve of 65 °C to 95 °C. Reactions were conducted on a CFX96 Real-time PCR Detection System (Bio-Rad). All data were analyzed with CFX Manager Software (Bio-Rad).

## Supplementary information

**Additional file 1.** Total proteins identified in maize leaves.

**Additional file 2.** Total differentially expressed proteins identified in maize leaves.

**Additional file 3.** Outline of the experiment design.

**Additional file 4.** Details of iTRAQ proteomics analysis methods.

**Additional file 5.** Primer sequences of DEPs encoding genes used for qRT-PCR.

## Data Availability

The datasets supporting the conclusions of this article are included within the article and its additional files.

## Abbreviations

ACN: Acetonitril
ANOVA: Analysis of variance
ATP: Adenosine triphosphate
*C*_*i*_: Intercellular CO_2_ concentration
CK: Ambient sunlight in the field
Cpr: Cysteine protease
DAPs: Differentially abundant proteins
DH605: Denghai 605
FA: Formic acid
FBPase: Fructose-bisphosphate aldolase
*g*_*s*_: Stomatal conductance
GSTs: Glutathione S-transferase
Hsp: Heat shock protein
iTRAQ: Isobaric tag for relative and absolute quantification
L: Supplementary lighting on cloudy daylight
LSD: Least significant difference
MDA: Malondialdehyde
MS: Mass spectrometry
PAR: Photosynthetically active radiation
*P*_*n*_: Photosynthetic rate
Prx: Peroxidase
PSI: Photosystem I
PSII: Photosystem II
qRT-PCR: Quantitative real time polymerase chain reaction
R6: Maturity stage
ROS: Reactive oxygen species
S: 60% Shading
SPAD: Chlorophyll soil–plant analyses development
*T*_*r*_: Transpiration rate
VT: Tasseling stage
VT20: 20 d after tasseling
VT40: 40 d after tasseling
