# Supplementary material for: Physiological and comparative proteomic analysis provides new insights into the effects of shade stress in maize (Zea mays L.)
Source: BMC Plant Biol. 2020 Feb 5;20:60. doi: 10.1186/s12870-020-2264-2 (PMC7003340; doi:10.1186/s12870-020-2264-2)
Supplement: Supplementary file 3 — Additional file 3. Outline of the experiment design. [file 12870_2020_2264_MOESM3_ESM.pdf]

Sample

**VT20S**	**VT20CK**	**VT20L**
**VT40S**	**VT40CK**	**VT40L**

Protein extraction and trypsin digestion

Sample groups	Labeling information
VT20S	114
VT20CK	115
VT20L	116
VT40S	117
VT40CK	118
VT40L	119

Note: three repetitions for each sample group

HPLC Fractionation

LC-MS/MS Analysis

Name	Identified	Quantified
Protein	3958	2745

Database Search

Bioinformatics Analysis
