# Supplementary material for: Physiological and comparative proteomic analysis provides new insights into the effects of shade stress in maize (Zea mays L.)
Source: BMC Plant Biol. 2020 Feb 5;20:60. doi: 10.1186/s12870-020-2264-2 (PMC7003340; doi:10.1186/s12870-020-2264-2)
Supplement: Supplementary file 4 — Additional file 4. Details of iTRAQ proteomics analysis methods. [file 12870_2020_2264_MOESM4_ESM.docx]

**Details of iTRAQ proteomics analysis methods**

**Sample preparation**

According to previous studies, we chose the VT20 and VT40 stages as two distinct phases during which to determine the proteins expression changes during the early and late stages of grain filling. We sampled five ear leaves from five plants at the center of each plot at VT20 and VT40. The middle portions of the leaves were collected and frozen in liquid nitrogen and stored at −80°C prior to analysis. Each treatment had three biological replicates (Additional file 1).

**Protein extraction**

Samples were ground into a fine powder in liquid nitrogen using a mortar and pestle. Then the cell powder was transferred to a 5 mL centrifuge tube and sonicated three times on ice using a high-intensity ultrasonic processor (Scientz) in lysis buffer (8 M urea, 2 mM EDTA, 10 mM DTT, and 1% protease inhibitor cocktail). The remaining debris was removed by centrifugation at 20,000g for 10 min at 4°C. Finally, the protein was precipitated with cold 15% TCA for 2 h at –20°C. After centrifugation at 4°C for 10 min, the supernatant was discarded. The remaining precipitate was washed three times with cold acetone. The protein was re-dissolved in buffer (8 M urea, 100 mM TEAB, pH 8.0) and the protein concentration was determined using the 2-D Quant kit according to the manufacturer’s instructions.

**Trypsin digestion and iTRAQ labeling**

The protein solution was reduced with 10 mM DTT at 37°C for 1 h and alkylated with 20 mM iodoacetamide at room temperature in darkness for 45 min. Then the protein sample was diluted by adding 100 mM TEAB to urea (concentration < 2M). Finally, trypsin was added at a 1:50 trypsin: protein mass ratio for the first digestion overnight and at 1:100 for a second 4 h digestion. Approximately 100 μg protein was digested with trypsin for each sample for the following experiments.

Following trypsin digestion, the peptide was desalted using a Strata X C18 SPE column (Phenomenex) and vacuum-dried. Peptide was reconstituted in 20 μl 0.5 M TEAB and processed according to the manufacturer’s protocol for the 8-plex iTRAQ kit (Additional file 1). Briefly, one unit of iTRAQ reagent (the amount of reagent required to label 100 μg protein) was reconstituted in 24 μL acetonitrile (ACN). Then the peptide mixtures were incubated for 2 h at room temperature and pooled, desalted, and dried by vacuum centrifugation.

**High-performance liquid chromatography fractionation**

Then the sample was fractionated into fractions by high-pH reverse-phase HPLC using the Agilent 300Extend C18 column (5 μm particles, 4.6 mm ID, 250 mm in length). Briefly, the peptides were separated into 80 fractions with a gradient of 2–60% acetonitrile in 10 mM ammonium bicarbonate (pH 10) for 80 min. Then the peptides were combined into 18 fractions and dried by vacuum centrifuging.

**Liquid chromatography–tandem mass spectrometry analysis**

Peptides were dissolved in 0.1% formic acid (FA) and directly loaded onto a reversed-phase pre-column (Acclaim PepMap 100, Thermo Scientific). Peptide separation was performed using a reversed-phase analytical column (Acclaim PepMap RSLC, Thermo Scientific). The gradient included an increase of 5% to 25% solvent B (0.1% FA in 98% ACN) over 24 min, 25% to 40% over 8 min, climbing to 80% over 3 min, and then holding at 80% for the last 3 min, all at a constant flow rate of 400 nL/min on an EASY-nLC 1000 UPLC system. The resulting peptides were analyzed using a Q Exactive^TM^ hybrid quadrupole-Orbitrap mass spectrometer (ThermoFisher Scientific).

The peptides were subjected to a nanospray ionization source followed by tandem mass spectrometry (MS/MS) in a Q Exactive^TM^ (Thermo) coupled online to the UPLC system. Intact peptides were detected in the Orbitrap at a resolution of 70,000. Peptides were selected for MS/MS using anormalized collision energy setting of 30, ion fragments were detected in the Orbitrap at a resolution of 17,500. A data-dependent procedure that alternated between 1 MS scan followed by 20 MS/MS scans was applied for the top 20 precursor ions above a threshold ion count of 10,000 in the MS survey scan with 30.0 s dynamic exclusion. The electrospray voltage applied was 2.0 kV. Automatic gain control was implemented to prevent overfilling of the Orbitrap, 5E4 ions were accumulated for the generation of MS/MS spectra. The m/z scan range for the MS scans was 350–1800. The fixed first mass was set at 100 m/z.

**Sequence database search and data analysis**

The resulting MS/MS data were processed using the Mascot search engine (v.2.3.0, Matrix Science, London, UK). Tandem mass spectra were searched against *Zea mays* database. The search was performed specifying Trypsin/P as a cleavage enzyme, allowing up to two missing cleavages. Mass error was set at 10 ppm for precursor ions and 0.02 Da for fragment ions. Carbamidomethyl on Cys was specified as a fixed modification and oxidation on Met was specified as a variable modification. iTRAQ-8-plex was selected in Mascot for protein quantification. The false discovery rate was adjusted to <1% and the peptide ion score was set at ≥20. We used CK samples from the same period as a reference, all other samples were compared to CK. To ensure the accuracy of quantitative results, we obtained quantitative protein information from at least two biological replicates before further analysis. The average of three biologic replicates was taken as the final protein abundance, and proteins with average protein abundance that changed by more than 1.2-fold in different stages (p ≤ 0.05) were defined as DAPs.

Functional annotations of DAPs species were performed using GO. Based on these annotations, proteins were classified into three categories: biological process, cellular component, and molecular function. Then we used the WoLF PSORT software to predict subcellular localization. We used the KEGG database to predict the main metabolic pathways and the DAPS biochemical signal transduction pathways. Statistical analyses were conducted using analysis of variance (ANOVA) in SPSS 20.0. We assessed differences among treatments using a least significant difference (LSD) test at a probability level of 0.05.
